# Proceedings of the American Association for the Advancement of Science

**Published:** 1849-09

**Authors:** 


PROCEEDINGS OF THE AMERICAN ASSOCIATION FOR THE ADVANCEMENT OF SCIENCE.

This association commenced its second meeting, at Cambridge, Mass., August, 1849. From the published proceedings, the meeting was one of unusual interest, and we have read them with more than ordinary gratification. We regret, however, that our limited space will permit us to give but a very meagre view of the chief topics before the association.

The meeting commenced its sessions, on the 14th day of August.—Professor Joseph Henry, the President of the Association, was present, and presided over its deliberations. In taking the chair, Prof. Henry made some explanatory remarks, on the objects of the association, which we subjoin, as indicative of the character of the proceedings. Professor Henry remarked: "We are not assembled to exhibit our knowledge of other men's thoughts, but to present to each other for consideration and friendly discussion, new truths—the results of original investigation—to inform each other of what we have done during the past year, and to ask assistance, advice and co-operation—to elicit, by intercourse and the collision of mind, new suggestions, to be proved or disproved during the next year. It is to be presumed, therefore, that the time of the association will not in any case be occupied with mere popular expositions of well known subjects."

In the narrow space to which we are limited, we can give but little more than the titles of the leading and most important subjects introduced to the association.

The first paper read, was on the *Aurora Borealis;* by Prof. Secchi, of Georgetown College. Its facts are very curious and interesting.

The second paper was read by Prof. Gray, and was devoted to a notice of that singular plant, called the *Polar plant,* or *Silphium Laciniatum.*

The fourth paper was prepared by Prof. Troost, on the *Crinoids.*—That gentleman has probably the largest and finest collection of Crinoids to be found in any cabinet, and we are pleased to learn that he is preparing a monograph upon their characteristics.

The seventh communication was a lecture, by Prof. Agassiz, "*On the structure of Coral Animals.*"

On the second day, papers were read on "*The Prime Meridian,*" by Lieut. Davis; on "*The results obtained in Geodysy by the application of the theory of the least squares,*" by Professor Bache; on "*Boltonite,*" by L. Saemann, Esq.,—the latter of which called forth some interesting remarks, from Prof. Silliman, Jr., recently elected to the chair of Chemistry in the Louisville Medical School. Prof. Agassiz also read papers on "*The zoological character of young mammalia,*" and "*The vegetable character of Xanthidium.*" Dr. Weightman read a communication on "*The Valerianate of Morphia,*" a salt of opium, supposed to possess many advantages over any other preparation of opium.

We are compelled to pass, in silence, many of the proceedings of the second day. On the third day of the session, Prof. Silliman, Jr., read a number of interesting analyses, made by him, to the association. Prof. Bache contributed an oral communication "*On the progress of the survey of the coast of the United States.*" Prof. Henry D. Rogers read an able paper "*On the origin of the drift, and of the lake and river terraces of the United States and Europe, with an examination of the laws of aqueous action connected with the inquiry.*" The theory of Prof. Rogers was ably supported, but it does not account for all the phenomena of the geological riddle which it undertakes to explain.

On the fifth day, Dr. Burnett read a valuable paper "*On some new points in the Morphology of cells, touching its analogy to that of the ovum.* This paper called forth an animated and instructive discussion.

Prof. Walter R. Johnson read a valuable paper "*On the economic values of American and British coals.*"

We regret that several of the papers, containing the report of the proceedings of the association, have failed to reach us, and we are, consequently, unable to refer fully to the regular order of the business.—We hope, however, to do justice to the subject, in our next number.

